# Ethanol extract of propolis and its constituent caffeic acid phenethyl ester inhibit breast cancer cells proliferation in inflammatory microenvironment by inhibiting TLR4 signal pathway and inducing apoptosis and autophagy

**DOI:** 10.1186/s12906-017-1984-9

**Published:** 2017-09-26

**Authors:** Huasong Chang, Yuehua Wang, Xusheng Yin, Xinying Liu, Hongzhuan Xuan

**Affiliations:** 1 School of Life Science, Liaocheng University, Liaocheng, 252059 China; 2 Institute of Apiculture and bee product quality inspection of Shandong Province, Taian, 271000 China

**Keywords:** Propolis, Caffeic acid phenethyl ester, Anti-inflammatory, Antitumor, Toll like receptor 4

## Abstract

**Background:**

Propolis and its major constituent – caffeic acid phenethyl ester (CAPE) have good abilities on antitumor and anti-inflammation. However, little is known about the actions of propolis and CAPE on tumor in inflammatory microenvironment, and inflammatory responses play decisive roles at different stages of tumor development. To understand the effects and mechanisms of ethanol-extracted Chinese propolis (EECP) and its major constituent - CAPE in inflammation-stimulated tumor, we investigated their effects on Toll-like receptor 4 (TLR4) signaling pathway which plays a crucial role in breast cancer MDA-MB-231 cell line.

**Methods:**

80% confluent breast cancer MDA-MB-231 cells were stimulated with 1 μg/mL lipopolysaccaride (LPS). Then the cells were divided for treatment by CAPE (25 μg/mL) and EECP (25, 50 and 100 μg/mL), respectively. Cell viability, nitric oxide (NO) production and cell migration were measured by sulforhodamine B assay, chemical method and scratch assay. The levels of TLR4, MyD88, IRAK4, TRIF, caspase 3, PARP, LC3B and p62 were investigated through western blotting. The expression of TLR4, LC3B and nuclear factor-κB p65 (NF-κB p65) were tested by immunofluorescence microscopy assay.

**Results:**

Treatment of different concentrations of EECP (25, 50 and 100 μg/mL) and CAPE (25 μg/mL) significantly inhibited LPS-stimulated MDA-MB-231 cell line proliferation, migration and NO production. Furthermore, EECP and CAPE activated caspase3 and PARP to induce cell apoptosis, and also upregulated LC3-II and decreased p62 level to induce autophagy during the process. TLR4 signaling pathway molecules such as TLR4, MyD88, IRAK4, TRIF and NF-κB p65 were all down-regulated after EECP and CAPE treatment in LPS-stimulated MDA-MB-231 cells.

**Conclusions:**

These findings indicated that EECP and its major constituent - CAPE inhibited breast cancer MDA-MB-231 cells proliferation in inflammatory microenvironment through activating apoptosis, autophagy and inhibiting TLR4 signaling pathway. EECP and CAPE may hold promising prospects in treating inflammation-induced tumor.

## Background

Propolis is a resinous material that honey bees collect from various plants, and it has been widely used in folk medicine, food or beverages to improve health and prevent diseases since ancient time for its numerous biological and pharmacological properties, such as antibacterial, antiviral, antitumor and anti-inflammatory activities [1]. Caffeic acid phenethyl ester (CAPE), one of the most important medicinal constituents of propolis, has varieties of important biological activities, including antioxidant, anti-inflammatory, and anti-cancer [2–4].

In recent years, the anti-inflammatory and antitumor actions of propolis and CAPE have been widely documented, and we also found Chinese propolis exhibited significant anti-inflammatory and antitumor effects in vivo and in vitro [5, 6], and indentified the bioactive components of Chinese propolis on antitumor activity [7]. However, little is known about the actions of propolis and its major active composition – CAPE on tumor in inflammatory microenvironment. Many researches demonstrate that inflammatory responses play decisive roles at different stages of tumor development, including initiation, promotion, malignant conversion, invasion, and metastasis [8]. Toll-like receptor 4 (TLR4) is well known for its host innate immunity, and recent evidence suggests that TLR4 is over-expressed in the majority of clinical breast cancer and involvement in breast cancer development and progression. Furthermore, the overactivation of TLR4 may cause immune response dysfunction, resulting in tumorigenesis. Thus, reducing expression of TLR4 could inhibit human breast cancer MDA-MB-231 cells proliferation and inflammatory cytokines secretion [9].

Inflammatory microenvironment plays an important role in tumor development, but little is known about the actions of propolis and its major active composition – CAPE on inflammation-mediated tumor. Thus, the goal of this study was to explore the effects of ethanol extract of Chinese propolis (EECP) and CAPE on lipopolysaccharide (LPS)-stimulated breast cancer MDA-MB-231 cells by testing TLR4 signaling pathway.

In addition, there is a high correlation between autophagy, inflammation and tumor [10–13]. Autophagy is a self-protective mechanism to maintain homestasis by breaking down the intracellular impaired protein or organelles, and it is considered as a two-edge sword having both anti- and pro-tumor functions at different stages of tumor development [14, 15], and more and more new insights strongly indicate that autophagy not only plays its classical role as a house-keeping mechanism, but also can be considered as crucial for induction and modulation of inflammatory reaction [16]. Thus, this study also aimed to evaluate whether propolis and its major constituent – CAPE modulated autophagy in LPS-stimulated breast cancer MDA-MB-231 cells.

## Methods

### Materials

Dulbecco’s Modified Eagle’s Medium (DMEM) was purchased from Gibco-BRL (USA). Fetal bovine serum (FBS) was from Hyclone Lab Inc. (USA). LPS (*Escherichia coli* 0111:B4), sulforhodamine B (SRB), prodium iodide (PI) and CAPE were from Sigma Co. (USA). Primary antibodies against TLR4, NF-κB p65, β-actin and secondary antibody (horseradish peroxidase) were from Santa Cruz Biotechnology (USA). Primary antibodies against MyD88, TRIF, IRAK4, LC3B, PARP and procaspase 3 were purchased from Cell Signaling Technology (USA). Secondary antibody for immunofluorescence, donkey anti-rabbit IgG Alexa Fluor-488 was purchased from Life Technologies (USA). Nitric oxide (NO) kit was from Nanjing Jiancheng Bioengineering Institute (China). All other reagents were ultrapure grade.

### Preparation of propolis ethanol extracts

Propolis used in the present study was Chinese propolis from Shandong Province of North China and the sample was collected from *A.mellifera* colonies from the wild, and it was unnecessary to gain permission for this prior to collection. The main plant origin was poplar (*Populus* sp.). Propolis used in the present experiment was the same as before and the extraction method was as used previously [6]. The ethanol-extracted Chinese propolis (EECP) had a brown color. The prepared propolis was stored under a dry condition at 4 °C.

### Total flavonoids measurement and HPLC analysis

Total flavonoids content of EECP was measured by the method of Chinese Standard (GB/T 20574–2006). The absorbance was read at 415 nm using an Ultraviolet Spectrophotometry.

HPLC analysis of EECP and CAPE was performed on a Century SIL C18 Eps column (250 mm × 4.6 mm I. D., 5 μm). The mobile phase consisted of methanol and 0.1% phosphoric acid in gradient elution mode (methanol: 0-8 min, 60%–70%; 8–30 min, 70%; 30–40 min, 70%–80%; 40–50 min). The flow rate of the mobile phase was kept at 1.0 mL/min, and the column temperature was kept at 28°C. The effluent was monitored by a photodiode array detector (PAD) at 280 nm.

### Cell culture

Breast cancer cell lines MDA-MB-231 was gifted by the Second Military Medical University of China. MDA-MB-231 cells was routinely cultured in DMEM supplemented with 10% (*v*/v) FBS and 100 U/mL of penicillin, 100 μg/mL streptomycin at 37 °C under humidified 95%–5% (v/v) air and CO_2_.

### Exposure of MDA-MB-231 cells to EECP

When the MDA-MB −231 cultures reached 80% confluence, the cells were divided for treatment: (a) culture in DMEM medium with 10% FBS with ethanol at <0.1% (*v*/v) (control); (b) the cells were stimulated with 1 μg/mL LPS but cultured in DMEM medium in the presence of 10% FBS with ethanol at <0.1% (*v*/v) (LPS); and (c) the cells were stimulated with 1 μg/mL LPS but culture in DMEM medium in the presence of 10% FBS with CAPE (25 μg/mL) and EECP (25, 50 and 100 μg/mL), respectively. EECP was dissolved in ethanol, with final concentration of ethanol in the culture medium <0.1% (v/v). The morphological changes of cells were observed under a phase contrast microscope (Nikon, Japan).

### Cell viability assay

Cells were seeded at the density of 4 × 10^4^/mL into 96-well plates and treated with different concentrations of EECP (25, 50 and 100 μg/mL) and CAPE (25 μg/mL) stimulated with LPS (1 μg/mL). At 24 and 48 h, SRB assay was used to determine cell viability. Briefly, cells were precipitated for 1 h at 4 °C with 100 μL 10% trichloroacetic acid and stained with SRB. The optical density was measured at 492 nm after reconstitution of the dye in 100 μL 10 mM Tris base. The viability (%) was expressed as (OD of treated group/OD of LPS group) × 100%. The viability of the LPS group was set at 100%.

### In vitro scratch assays

Cells were grown to 80% confluence in a 24-well plate, the monolayers were scratched with a plastic tip to create a straight-line cell-free scratch, washed by 1 × PBS to remove floating cell debris, and then incubated in medium in the absence or presence of different concentrations of EECP and CAPE stimulated with LPS (1 μg/mL) for 48 h. The scratch area was marked and photographed in every 12 h. Cell migration into the wound surface was determined under a TE2000S inverted microscope (Nikon, Japan). Migrated cells across the scratched lines were counted by Image-Pro Plus software (USA).

### NO production measurement

The cell medium was collected and centrifuged at 48 h, then dispensed and stored at −80 °C until tested. The generation of NO was assayed by a NO measurement kit.

### Immunofluorescence microscopy assay

Immunofluorescence assay was performed by the method [17]. MDA-MB-231 cells treated for 48 h were fixed in 4% paraformaldehyde (*w*/*v*) for 15 min at room temperature and blocked in 5% donkey serum (*v*/v). After adding the primary and second antibodies (FITC-IgG), nuclei were counterstained with PI. A laser scanning confocal microscope (Olympus FV1200, Japan) was used for fluorescence detection. Analysis was made by the Image-Pro Plus software (USA). Images are representative of three independent experiments.

### Western blotting analysis

Western blotting assay was performed by a previous method [17]. Cells were washed three times with cold phosphate-buffer saline and lysed in lysis buffer with protease inhibitors at ice. Thirty micrograms of protein were separated by 12% sodium dodecyl sulfate polyacrylamide gel electrophoresis (SDS-PAGE) and electro-blotted to a polyvinylidene difluoride (PVDF) membrane using a semi-dry blotting apparatus (Bio-Rad, USA). The bands are visualized using an enhanced chemiluminesence detection kit (Thermo Electron Corp., USA). β-actin was used as loading control.

### Statistical analysis

Data are from at least three independent experiments and expressed as means ± S.E.M. Statistical analysis involved the paired Student *t* test and ANOVA with SPSS Ins (PASW Statistics 18). Differences were considered statistically significant at *P* < 0.05.

## Results

### Total flavonoids content of EECP and HPLC assay

**Fig. 1:**
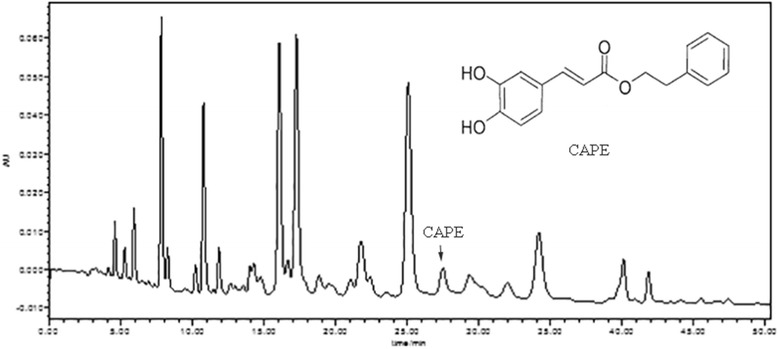
HPLC chromatograms of ethanol-extracted Chinese propolis (EECP) and caffeic acid phenethyl ester (CAPE)

Propolis used in the present study was Chinese propolis from Shandong Province, and the main plant origin was poplar (*Populus* sp.). Total flavonoids content of EECP was 22.68%, and the content of CAPE in EECP was 0.11% (Fig. 1).

### EECP and CAPE decreased LPS-stimulated MDA-MB-231 cells proliferation

**Fig. 2:**
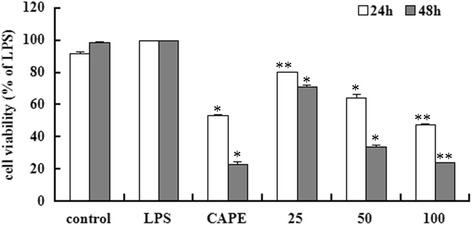
EECP and CAPE decreased LPS-stimulated MDA-MB-231 cells proliferation at 24 and 48 h. CAPE, cells treated with CAPE at 25 μg/mL. 25, 50 and 100 μg/mL, cells treated with EECP at 25, 50 and 100 μg/mL, respectively. (^*^ *P* < 0.05, ^**^ *P* < 0.01 vs control, *n* = 3). Data are means ± S.E.M

Cell viability was analyzed by SRB assay and the results showed that CAPE and different concentrations of EECP exhibited an obviously inhibitory effect on the proliferation of MDA-MB-231 cells stimulated by LPS in a time- and dose-dependent manner. And the inhibitory effect of CAPE (25 μg/mL) was similar with EECP 50–100 μg/mL (^*^ *P* < 0.05, ^**^ *P* < 0.01; Fig. 2).

### EECP and CAPE inhibited LPS-stimulated MDA-MB-231 cells migration

**Fig. 3:**
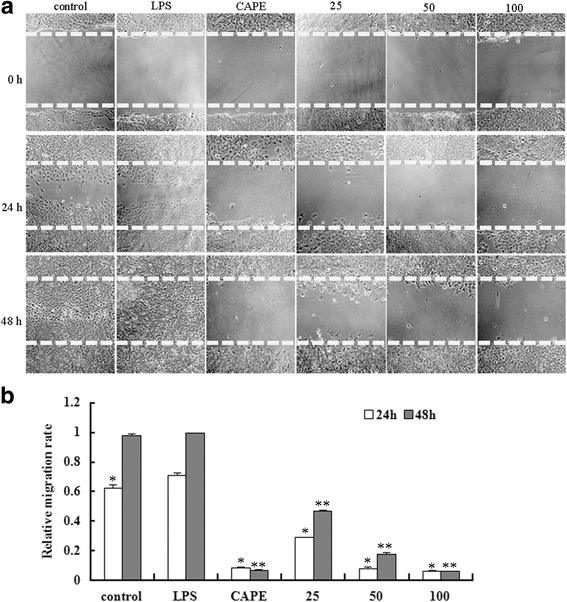
EECP and CAPE inhibited LPS-stimulated MDA-MB-231 cells migration. **a** Cell migration micrographs obtained under a phase contrast microscope at 0, 24 and 48 h (×100). **b** Relative levels of cell migration. (^*^ *P* < 0.05, ^**^ *P* < 0.01 vs control, *n* = 3). Data are means ± S.E.M

The migration ability of MDA-MB-231 cells was significantly increased upon LPS treatment. CAPE and different concentrations of EECP significantly inhibited cell migration in a dose-dependent manner at 24 and 48 h (^*^ *P* < 0.05, ^**^ *P* < 0.01; Fig. 3).

### EECP and CAPE inhibited NO production in LPS-stimulated MDA-MB-231 cells

**Fig. 4:**
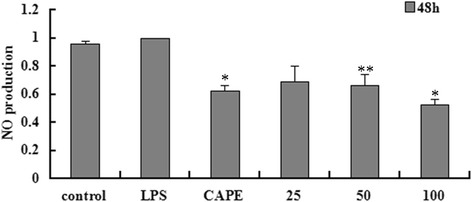
EECP and CAPE inhibited NO production at 48 h. (^*^ *P* < 0.05, ^**^ *P* < 0.01 vs control, n = 3). Data are means ± S.E.M

The production of NO in cell supernate was obviously decreased by CAPE and different concentrations of EECP treatment at 48 h. (^*^ *P* < 0.05, ^**^ *P* < 0.01; Fig. 4).

### EECP and CAPE induced apoptosis and autophagy in LPS-stimulated MDA-MB-231 cells

**Fig. 5:**
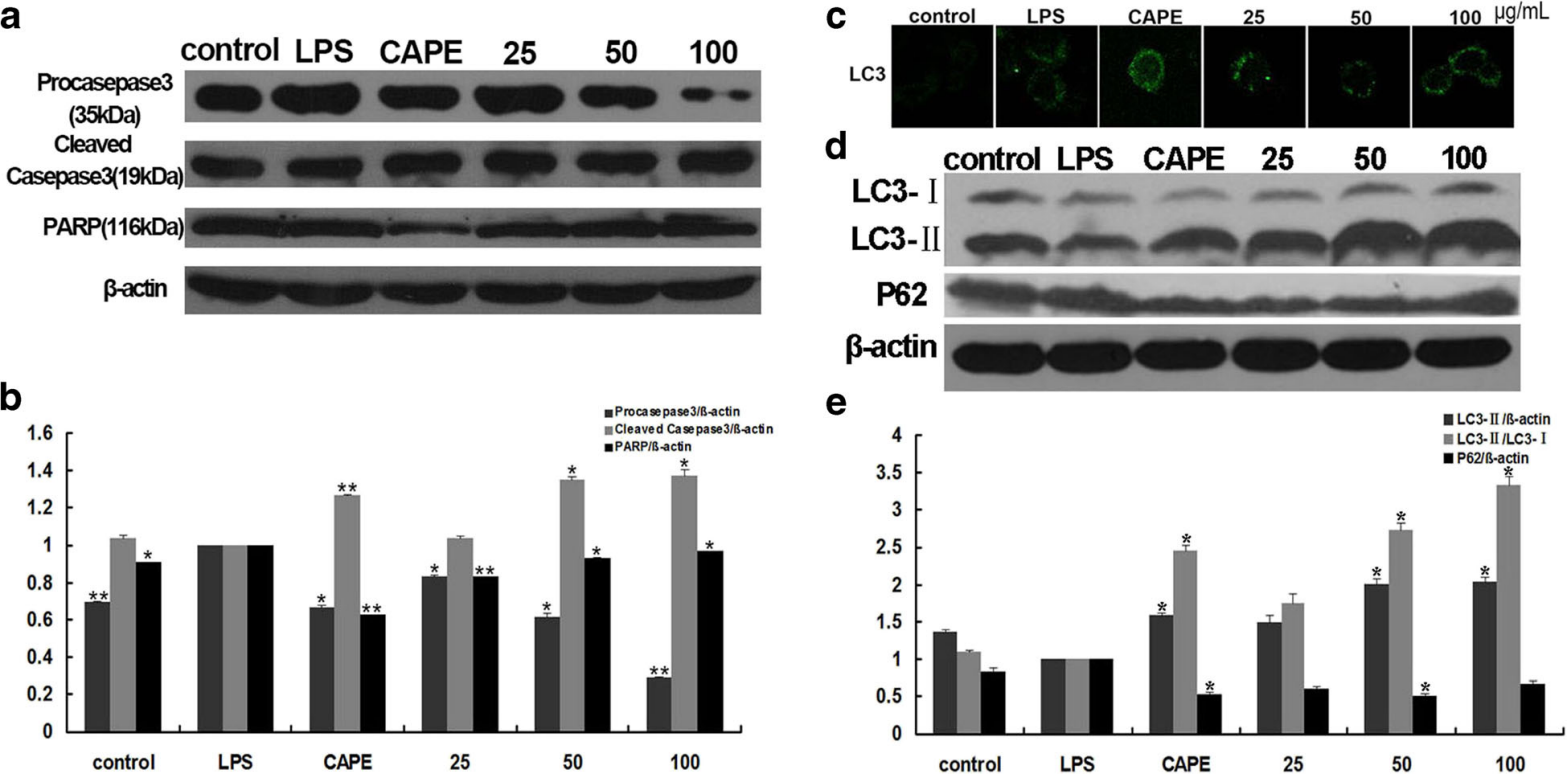
EECP and CAPE induced apoptosis and autophagy in LPS-stimulated MDA-MB-231 cells. **a** The expression of caspase 3, procaspase 3 and PARP were detected by western blotting at 24 h. **b** Quantification of relative expression of procaspse 3, caspase 3 and PARP. **c** Cells were stained with anti-LC3B antibody for immunostaining. Immunofluorescence graphs showed an increase of endogenous punctuate LC3B. **d** The expression of LC3B and p62 were detected by western blotting at 24 h. **e** Quantification of relative expression of LC3-II, LC3-II/ LC3-Iand p62 in LPS-stimulated MDA-MB-231 cells. (^*^ *P* < 0.05, ^**^ *P* < 0.01 vs control, n = 3). Data are means ± S.E.M

We first tested the effects of EECP and CAPE on apoptosis in LPS-stimulated MDA-MB-231 cells. EECP (25–100 μg/mL) and CAPE (25 μg/mL) obviously activated caspase 3 and PARP, the executor of apoptosis (Fig. 5a). We then tested whether EECP and CAPE affected autophagy during the process. It is acceptable that the enhancement of the conversion of LC3-I to LC3-II, up-regulation of LC3-II expression and the degration of p62 are the credible markers of the autophagosome in mammalian cells [18, 19]. Distribution of LC3 was detected with an immunostaining assay. When challenged with EECP (25–100 μg/mL) and CAPE (25 μg/mL), cells stained with anti-LC3 antibody showed a distinctively punctuate pattern compared with LPS group (Fig. 5c). Furthermore, EECP and CAPE treatment obviously elevated the level of LC3-II and the ratio of LC3-II/LC3-I. The level of p62 significantly decreased after EECP and CAPE treatment in LPS-stimulated MDA-MB-231 cells at 24 h (^*^ *P* < 0.05, ^**^ *P* < 0.01; Fig. 5d).

### EECP and CAPE regulated the levels of TLR4, MyD88, IRAK4 and TRIF in LPS-stimulated MDA-MB-231 cells

**Fig. 6:**
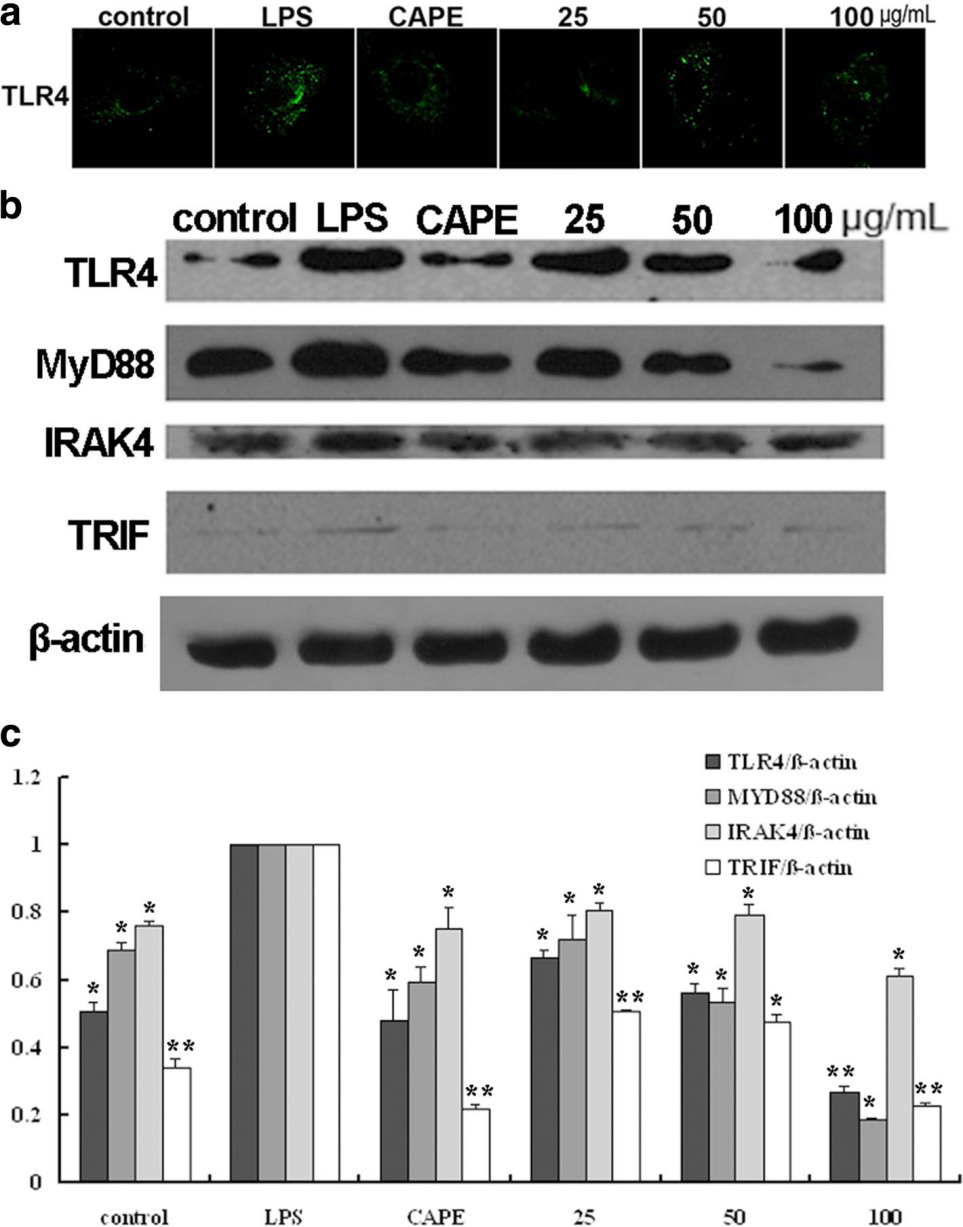
EECP and CAPE regulated the levels of TLR4, MyD88, IRAK4 and TRIF. **a** Cells were treated with EECP and CAPE for 24 h, respectively. Cells were stained with anti-TLR4 antibody. Immunofluorescence graphs showed a decrease of TLR4 level. **b** The expression of TLR4, MyD88, IRAK4 and TRIF in LPS-stimulated MDA-MB-231 cells were detected by western blotting at 48 h. **c** Quantification of relative expression of TLR4, MyD88, IRAK4 and TRIF in LPS-stimulated MDA-MB-231 cells. (^*^ *P* < 0.05, ^**^ *P* < 0.01 vs control, n = 3). Data are means ± S.E.M

It is reported that TLR4 is over-expressed in the majority of clinical breast cancer and involvement in breast cancer development and progression. In order to investigate whether EECP and CAPE affected TLR4 signaling pathway, we first tested the level of TLR4 by western blotting and immunofluorescence analysis. Cells treated with EECP and CAPE exhibited lower florescence intensity compared with LPS group (Fig. 6a), and protein level of TLR4 evidently decreased after EECP and CAPE treatment. Then we further tested the downstream signal molecules of TLR4 such as MyD88, IRAK4 and TRIF. The results showed that CAPE and different concentrations of EECP significantly decreased the expression of MyD88, IRAK4 and TRIF (^*^ *P* < 0.05, ^**^ *P* < 0.01; Fig. 6b).

### EECP and CAPE regulated the levels of NF- κB p65 in LPS-stimulated MDA-MB-231 cells

**Fig. 7:**
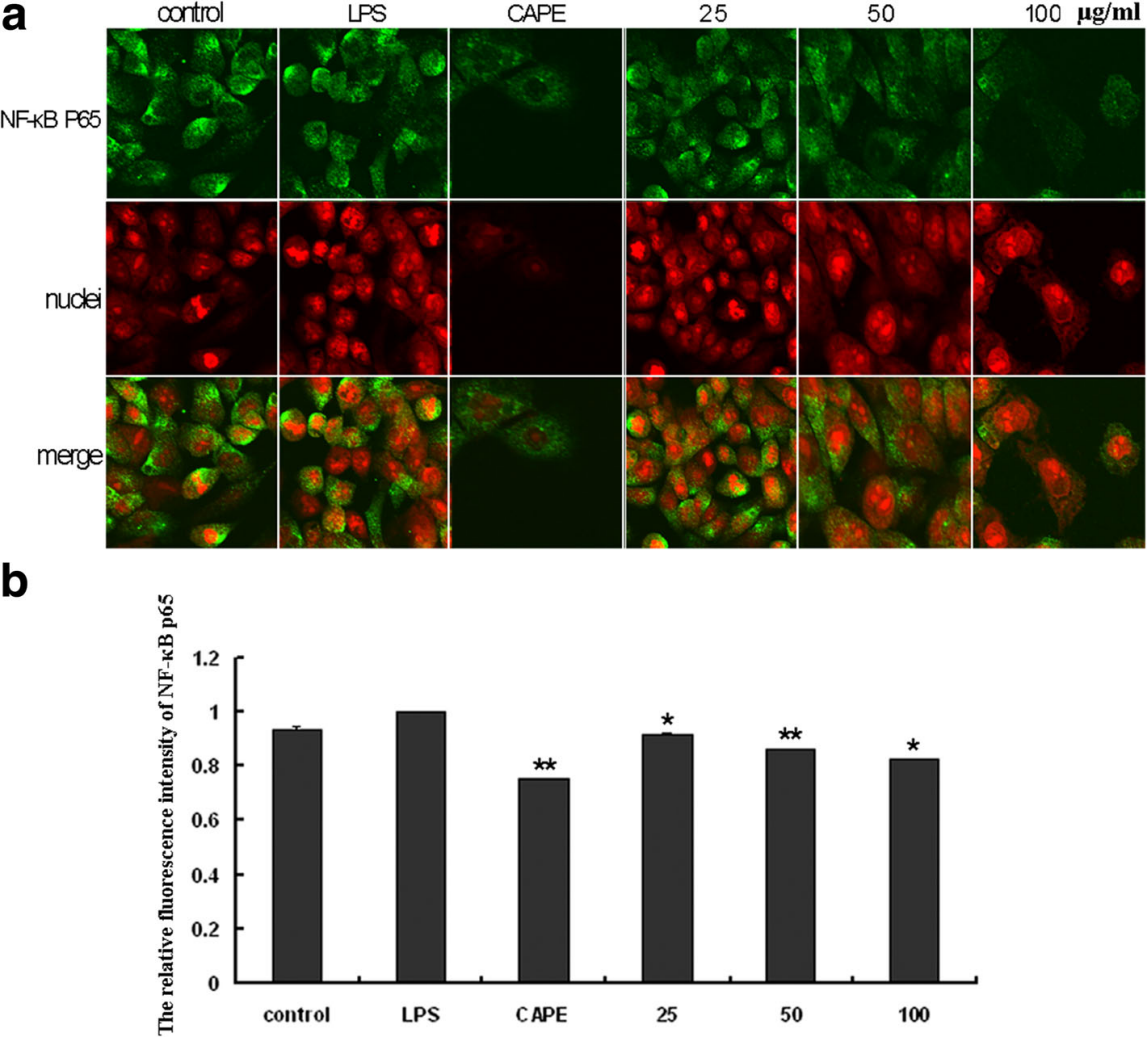
EECP and CAPE regulated the levels of NF- κB p65. **a** Fluorescent micrographs obtained at 48 h (×400). **b** The relative fluorescence intensity of NF-κB p65 in LPS-stimulated MDA-MB-231 cells. (^*^ *P* < 0.05, ^**^ *P* < 0.01 vs control, n = 3). Data are means ± S.E.M

CAPE and different concentrations of EECP significantly down-regulated NF-κB p65 level in a dose-dependent manner by immunofluorescence assay in LPS-stimulated MDA-MB-231 cells. Furthermore, the translocation of NF-κB p65 from cytoplasm to nuclei was also inhibited by CAPE and EECP treatment. (^*^ *P* < 0.05, ^**^ *P* < 0.01; Fig. 7).

## Discussion

Propolis is a plant-derived substance collected by honeybees from various sources, and there are more than 600 constituents in propolis, mainly flavonoids and phenolic acids [20]. Our previous study showed propolis had good antitumor effects on different cancer cells such as the human breast cancer cells (MCF-7 and MDA-MB-231), lung cancer cells (A549), human cervical carcinoma cell (HeLa) [6]. And the antitumor bioactive constituents are flavonoids and esters [7]. In addition, propolis exhibit excellent antiinflammatory activities in macrophages (Raw 264.7), ox-LDL stimulated HUVECs and intestinal epithelial Caco-2 cells by modulating key inflammatory mediators of mRNA transcription, inhibiting the production of specific inflammatory cytokines, blocking the activation of nuclear factor NF-κB and activating AMPK and ERK signaling pathway [5, 21, 22].

Rudolf Virchow provided the first indication of a possible link between inflammation and cancer in the nineteenth century. And in recent years, numerous studies demonstrated that inflammation plays a critical role in tumorigenesis [23–25]. Accumulating evidence indicated that propolis and CAPE had good abilities in antitumor and anti-inflammatory. Here, we are the first to evaluate the actions of propolis and its major active composition – CAPE on tumor in inflammatory microenvironment, and we found propolis and CAPE induced autophagy and attenuated inflammatory signaling cascade in LPS-stimulated human breast cancer MDA-MB-231 cells, then depressed the proliferation of MDA-MB-231 cells.

TLR4 is well known for its host innate immunity. However, recent evidence shows that TLR4 is expressed in a wide variety of tumors such as liver cancer, lung cancer, breast cancer, gastrointestinal cancer, pancreatic cancer. Moreover, there is growing evidence that TLR4 activation appears to act as a double-edged sword in cancers, that is, TLR4 activation has been linked to both cancer inhibition and growth [26]. Yang et al. (2010) reported that ten TLRs were expressed in MDA-MB-231 cells, TLR4 expression was the highest among all the TLRs, and they also demonstrated that knockdown of TLR4 could actively inhibit proliferation and survival of breast cancer cells and RNAi-directed targeting of TLR4 may be a beneficial strategy for breast cancer therapy [27]. In present study, we found EECP and CAPE obviously attenuated TLR4 level in LPS-stimulated MDA-MB-231 cells, which provided novel insights into the potential application of propolis on antitumor in inflammatory microenvironment.

The TLR4 signaling pathway includes MyD88-dependent and MyD88-independent pathways [28]. Both pathways can activate NF-kB to release cytokine. We evaluated the MyD88-dependent signaling pathway molecules such as MyD88, IRAK4 expressions and TRIF which was involved in MyD88-independent pathway by western blotting assay. Our results suggested that Chinese propolis and its major constituent – CAPE inhibited TLR4 signaling pathway molecules such as TLR4, MyD88, IRAK4, TRIF and NF- kB p65, which might be one of the major causes for Chinese propolis and CAPE to inhibit breast cancer cell proliferation and survive. Based on these results, we deduced that TLR4 signaling pathway might be the target molecule for Chinese propolis and CAPE to inhibit breast cancer cell proliferation in inflammatory-mediated tumor microenvironment.

Autophagy is an evolutionarily conserved catabolic pathway involved in several physiological processes including cell metabolism, cell survival and host defense [29, 30]. More and more new insights strongly indicate that autophagy also can modulate host defense. Despite the increasing knowledge about the role of autophagy in modulation of inflammation, little is known about the involvement of the autophagy-related SQSTM1-like receptors for the modulation of inflammation [13]. Here we found that Chinese propolis and its major active constituent – CAPE promoted autophagy and inhibited TLR4 signaling pathway to prevent LPS-stimulated breast cancer MDA-MB-231 cells proliferation although the crosstalk between autophagy and inflammation was not known.

Apoptosis was another major cause for Chinese propolis and CAPE to inhibit LPS-stimulated MDA-MB-231 cells survival. We and other researchers all reported that propolis could induce cancer cells apoptosis [6, 31]. Here we also found that Chinese propolis and CAPE activated caspase 3- the executor of apoptosis in LPS-stimulated breast cancer cells, which might be induced by activating autophagy and depressing TLR4 signaling pathway.

NO, a physiological signaling molecule, is involved in many cellular functions, including cell proliferation, survival and death. A recent study suggested that LPS/TLR4-induced signaling cascades leads to inducible nitric oxide synthase (iNOS) induction, and inhibition of iNOS might be as a novel effective target therapy against triple-negative breast cancer [32]. In present study, we found Chinese propolis and CAPE obviously inhibited the production of NO, which might inhibit MDA-MB-231 cells survival.

## Conclusion

Based on our finding, propolis and its major component – CAPE could inhibit the proliferation and migration of the TLR4 positive breast cancer MDA-MB-231 cell line in inflammatory microenvironment by activation of apoptosis, autophagy and inhibition of TLR4 signaling pathway. These results provided new insights into the molecular mechanisms underlying the beneficial effects of propolis and CAPE on antitumor in inflammatory microenvironment. However, there are still many problems to be further studied. For example, the crosstalk between autophagy and TLR4 signaling pathway, and the effects of propolis and its major constituents on specific inflammatory cytokines secretion in inflammation-mediated tumor are still not known.

## Abbreviations

CAPE: Caffeic acid phenethyl ester
EECP: Ethanol extract of Chinese propolis
FBS: Fetal bovine serum
iNOS: inducible nitric oxide synthase
LPS: Lipopolysaccaride
NF-κB p65: Nuclear factor-κB p65
NO: Nitric oxide
PVDF: Polyvinylidene difluoride
SDS-PAGE: Sodium dodecyl sulfate polyacrylamide gel electrophoresis
SRB: Sulforhodamine B
TLR4: Toll-like receptor 4
